# Combination of *Lactobacillus plantarum* improves the effects of tacrolimus on colitis in a mouse model

**DOI:** 10.3389/fcimb.2023.1130820

**Published:** 2023-03-13

**Authors:** Wei Lv, Di Zhang, Tian He, Yingying Liu, Limei Shao, Zhongping Lv, Xiaoping Pu, Yufang Wang, Ling Liu

**Affiliations:** ^1^ Department of Gastroenterology, West China Hospital, Sichuan University, Chengdu, Sichuan, China; ^2^ Department of Urology, Beijing Chaoyang Hospital, Capital Medical University, Beijing, China; ^3^ Technology Research Institute of Shuxi Condiments of Sichuan Cuisine Co. LTD, Chengdu, Sichuan, China

**Keywords:** colitis, tacrolimus, gut microbiome, *Lactobacillus plantarum*, bile acid

## Abstract

The gut microbiome has been considered to play an important role in inflammatory bowel disease (IBD). Our previous study reported that tacrolimus-altered gut microbiota elicited immunoregulatory effects in both colonic mucosa and circulation, contributing to an increased allograft survival rate in mice. Here, we aimed to observe the changes in the tacrolimus-induced microbiome in a dextran sulfate sodium (DSS)-induced colitis mouse model and explore the possibility and efficacy of combination therapy with tacrolimus and the microbiome on colitis. Mice were divided into the control, DSS, tacrolimus monotherapy and tacrolimus plus *Lactobacillus plantarum* 550 (*Lacto*)-treated groups. The body weight, stool consistency, hematochezia and survival of mice were observed daily. Total RNA from colonic mucosa was extracted and subjected to transcriptome sequencing. Cecal contents were collected and the 16S rRNA sequencing was performed to characterize the gut microbiome and the ultrahigh- performance liquid chromatography-MS/MS (UHPLC-MS/MS) was used for targeted quantification of bile acids. The results confirmed that tacrolimus significantly ameliorated DSS-induced colitis in mice. Beneficial alterations of the gut microbiome characterized by a remarkable expansion of the genus *Lactobacillus* were induced by tacrolimus treatment. Oral supplementation with *Lacto* further improved the tacrolimus-mediated suppression of body weight loss in colitis, while the survival time of mice was further prolonged and the inflammation of colonic mucosa was obviously relieved. The immune and inflammation-related signaling pathways, including IFN-γ and IFN-α response, allograft rejection, IL2 STAT5 signaling and the inflammatory response pathways, were further downregulated in the tacrolimus plus *Lacto* cotreatment group. Cotreatment also improved the diversity of the gut microbiome and rescued the concentration of taurochenodeoxycholic acid (TCDCA) in colitis. The latter was positively correlated with the abundance of *Lactobacillus* but negatively related to the disease activity index score. Overall, our results indicated that *Lactobacillus plantarum* promoted the therapeutic effect of tacrolimus in experimental colitis, offering a promising strategy to combine tacrolimus and *Lactobacillus* in the treatment of colitis patients.

## Highlights

1. Tacrolimus induced beneficial alterations in the gut microbiome in DSS-induced colitis, characterized by a remarkable expansion of the genus_*Lactobacillus.*
2. Compared with tacrolimus monotherapy, oral supplementation with *Lactobacillus plantarum* further ameliorated colitis and inhibited the proinflammatory signaling pathways in mice.
3. The combination of tacrolimus and *Lactobacillus plantarum* improved the diversity and composition of the gut microbiome as well as bile acid metabolism in colitis mice.

## 1 Introduction

Inflammatory bowel disease (IBD), encompassing Crohn’s disease (CD) and ulcerative colitis (UC), is a group of chronic and relapsing inflammatory disorders that causes intestinal lesions and even extraintestinal manifestations (Wright et al., 2018). In the past decade, the incidence of IBD has significantly increased in newly industrialized countries or urbanized countries in Asia (Ng et al., 2017; Ng et al., 2019). This leads IBD to be a public health challenge worldwide. Considerable evidence suggests that the pathogenesis of IBD is characterized by an inappropriate and persistent mucosal immune response, caused by genetic and environmental factors (Caruso et al., 2020). The gut microbiome has been considered as a pivotal cause of the occurrence and development of IBD (Honda and Littman, 2012; Caruso et al., 2020). Dysbiosis in IBD manifests as a decrease in commensal bacterial diversity and alterations in the gut microbiota composition (Honda and Littman, 2012; Caruso et al., 2020). The finding that the genetically susceptible mice fail to develop colitis under germ-free conditions, and the effectiveness of antibiotics and probiotics in IBD therapy further support the important role of the gut microbiome. (Podolsky, 2002; Caruso et al., 2020; Lee and Chang, 2021).

Tacrolimus (Tacro), a commonly used immunosuppressant in the transplantation area, is also recommended in the treatment of refractory IBD patients with impressive efficacy (Baumgart et al., 2006; Rodríguez-Lago et al., 2020). With the advantages of high oral bioavailability and the potent inhibitory effects on activated T-cells(Fellermann et al., 2002; Komaki et al., 2016), FK has been selectively utilized to induce remission in corticosteroid-refractory UC and refractory fistulizing perianal CD in recent years (Rodríguez-Lago et al., 2020; Gordon et al., 2022). However, due to the relatively narrow therapeutic window and dose-dependent side effects such as neurotoxicity, nephrotoxicity, metabolic disorders and opportunistic infection (Baumgart et al., 2006), it is recommended that tacrolimus be used as a bridging strategy or for short-term induction of remission in IBD (Rodríguez-Lago et al., 2020). Further studies are needed to shed light on the strategy that reduces the side effects and enhances the efficacy of tacrolimus in IBD.

Conventionally, tacrolimus exerts its immunosuppressive functions by binding to the FK506-binding protein to inhibit calcineurin phosphatase and block the transcription and secretion of cytokines, particularly interleukin (IL)-2, which leads to the suppression of T lymphocyte activation and proliferation (Thomson et al., 1995; Chow and Leong, 2007). In recent years, studies by our group and other groups have reported that tacrolimus alters the composition and bacterial taxa of the gut microbiota (Jiang et al., 2018; Zhang et al., 2018). We have demonstrated a significant expansion of *Allobaculum, Bacteroides* and *Lactobacillus* in tacrolimus-treated mice (Zhang et al., 2018). Innovatively, our study reported that the tacrolimus-induced microbiota elicited immunoregulatory effects in both colonic mucosa and circulation, causing a significantly increased proportion of CD4^+^CD25^hi^FoxP3^+^ regulatory T cells in peripheral blood mononuclear cells, mesenteric lymph nodes and colonic mucosa (Zhang et al., 2018). This suppressive function of tacrolimus–induced gut microbiota was further confirmed in a skin transplant mouse model. Furthermore, our study confirmed that tacrolimus-induced disorders of glucose metabolism were partially associated with the gut microbiota, and oral supplementation with butyrate might prevent and reverse tacrolimus-induced hyperglycemia in mice (Jiao et al., 2020). These data raise the possibility of combining immunosuppressive agents and the gut microbiome for the treatment of immune diseases. However, to our knowledge, the composition and function of the tacrolimus-induced microbiome in the colitis model and the underlying mechanism remain unclear.

In this study, we evaluated the role of tacrolimus and its impact on the gut microbiota, immune response and bile acid profiles in dextran sulfate sodium (DSS)-induced colitis mouse model. Furthermore, the combination of tacrolimus and the gut microbiome might be considered as a prospective strategy for the treatment of colitis patients in the clinic.

## 2 Materials and methods

### 2.1 Animal studies

The animal experiments were conducted in two different centers (West China Hospital and Chaoyang Hospital) under the same conditions to confirm the reliability of the results. Six-week-old male C57BL/6 mice were obtained from Beijing Vital River Laboratory Animal Technology Corporation Ltd. and the Experimental Animal Center of Sichuan University. All animals were housed in specific pathogen-free (SPF) conditions under constant temperature and humidity with a 12 h light-dark cycle. Standard diet and sterile water were supplied ad libitum during the initial 7-day adaptation period. All animal procedures were approved by the Animal Ethics Committees of Beijing Chao-Yang Hospital and the Animal Ethics Committee of West China Hospital of Sichuan University (Ref. No. 20211277A).

### 2.2 Dextran sulfate sodium-induced colitis model and treatment

Mice were randomly divided into different groups of 6-10 animals each before the start of the experiment. Colitis was induced by adding 2.5% (w/v) dextran sulfate sodium (DSS, M.W: 36000-50000, MP Biomedicals, Canada) to the drinking water for 7 days, then replaced with sterile water. Tacrolimus (Tacro, Astellas Ireland Corporation Ltd, Shenyang, China) at different dosages (0.1 mg/kg, 1 mg/kg or 10 mg/kg) was administered *via* oral gavage in sterile water once a day and maintained until the end of experiments. *Lactobacillus plantarum* 550 (*Lacto*) in powder form was isolated from pickle and provided as a gift from Technology Research Institute of Shuxi Condiments of Sichuan Cuisine Corporation Ltd (Chengdu, China), 1×10^8^ CFU in 200 μL sterile water/mouse/day was given daily *via* oral gavage 3 days before DSS administration and maintained until the end of experiments. Mice in the control group were administered an equal volume of sterile water by gavage. Body weight and water consumption were recorded daily. The blood in the stool was detected by Pyramidon semiquantitative assay using a Fecal Occult Blood test kit (BA2020B, BaSO, Zhuhai, China) and recorded as “-”, “+”, “++”, “+++”, or “++++”. The disease activity index (DAI) was evaluated by scoring the body weight loss (%), stool consistency and blood in feces as described in the literature(Jang et al., 2019), with minor modifications (**Supplementary Table 1**). Mice were sacrificed at the end of the treatment, and the colon lengths were measured. Cecal contents were collected, snap frozen and kept at -80 °C for 16S rRNA sequencing and targeted bile acid metabolomics assays. Colonic mucosa was isolated and frozen until transcriptomics analysis. Sections of the proximal and distal colon were fixed in 4% paraformaldehyde and stained with hematoxylin and eosin (H&E). Histological scores were calculated according to the criteria described in the literature (a score from 0 to 4 was given for each colon segment [proximal or distal] based on the severity of inflammation, and a combined score [proximal and distal] provided a total colonic histological score per mouse) (Liu et al., 2017).

### 2.3 16S rRNA amplicon sequencing

The total genomic DNA was extracted from cecal contents of mice using the TGuide S96 Magnetic Soil and Stool DNA Kit (TIANGEN Biotech Corporation Ltd, Beijing, China) according to the manufacturer’s instructions. The full-length 16S rRNA gene was amplified with universal primers (27F: 5’-AGRGTTTGATYNTGGCTCAG-3’; 1492R:5’-TASGGHTACCTTGTTASGACTT-3’) under the following conditions: 95 °C for 2 min; 25 cycles of 98 °C for 10 s, 55 °C for 30 s, and 72 °C for 90 s; and a final step at 72°C for 2 min. The purified PCR products were sequenced on the PacBio platform by Biomarker Technologies Corporation (Beijing, China). The circular consensus sequencing (CCS) reads were generated from the corrected original subreads by SMRT Link (version 8.0) following the setting parameters: minPasses ≥ 5 and minPredictedAccuracy ≥ 0.9. After barcode and primer identification followed by chimera removal, high-quality reads were obtained and clustered as operational taxonomic units (OTUs) with similarity over 97% using USEARCH (version 10.0). Species annotation and taxonomic and diversity analyses were conducted with QIIME2 (version 2020.06). Chao1 and Shannon indexes were used to reflect alpha diversity, while nonmetric multidimensional scaling (NMDS) analysis was carried out to show the dissimilarity of beta diversity. The vegan package in R language was applied for ANOSIM analysis to measure the significant differences in beta diversity between groups. Linear discriminant analysis effect size (LEfSe) analysis with LDA score > 3 and statistical analysis of metagenomic profiles (STAMP) were performed to identify the significantly different bacteria between groups at the genus level.

### 2.4 Bile acid targeted metabolomic analysis

The high-throughput targeted quantification of bile acids in mice cecal contents was performed at Biomarker Technologies Corporation (Beijing, China) by UHPLC-MS/MS. Aliquots (25 mg) from 35 samples were weighed and added 1000 μL of precooled extract solution containing 0.1% formic acid and isotopically labeled internal standard mixture (acetonitrile-methanol-water, 2:2:1) was added per tube. After vortexing for 30 s, the mixture was homogenized at 35 Hz for 4 min, followed by sonication for 5 min in an ice-water bath. The latter 9-min circle was repeated three times, and then the tubes were incubated at -40°C for 1 h and centrifuged at 12000 rpm and 4°C for 15 min. The resulting supernatants were collected for UHPLC-MS/MS analysis.

An UHPLC System (Vanquish, ThermoFisher Scientific) equipped with a Waters ACQUITY UPLC BEH C18 column (150 *2.1 mm, 1.7 μm, Waters) was used for chromatographic separation. Mobile phase A included 1 mmol/L ammonium acetate and 0.1% acetic acid in water, and the mobile phase B was acetonitrile. The column temperature was set at 50°C, while the autosampler temperature was maintained at 4°C. The injection volume was 1 μL. Mass spectrometry in parallel reaction monitoring (PRM) mode was carried out by a Q Exactive HFX mass spectrometer (Thermo Fisher Scientific). The ion source parameters were as follows: spray voltage = +3500/-3100 V, sheath gas (N2) flow rate = 40, aux gas (N2) flow rate = 15, sweep gas (N2) flow rate = 0, aux gas (N2) temperature = 350°C, capillary temperature = 320°C.

A total of 39 bile acids were identified in this study (detailed in **Supplementary Table 2**). The calibration standard solutions were diluted stepwise with a dilution factor of 2 before being subjected to UHPLC-PRM-MS analysis. The final concentration (cF, nmol/L) of the samples measured was obtained by multiplying the directly calculated concentration (cC, nmol/L) output from the system by the dilution factor. The targeted metabolite concentration (cM, nmol/kg) in the tissue samples was calculated according to the following formula (VF: final volume of sample; m: sample mass): $C_{M}[nmol\cdot kg^{-1}]=\frac{C_{F}[nmol\cdot L^{-1}]\cdot V_{F}[\mu L]}{m[mg]}$

### 2.5 Transcriptome analysis

Total RNA from mice colonic mucosa was extracted using the mirVana miRNA Isolation Kit (Ambion) following the manufacturer’s protocol. RNA integrity was assessed by an Agilent 2100 Bioanalyzer (Agilent Technologies, Santa Clara, CA, USA). Samples with an RNA integrity number (RIN) ≥ 7 were subjected to the subsequent analysis. The TruSeq Stranded mRNA LT Sample Prep Kit (Illumina, San Diego, CA, USA) was used to construct the libraries. Transcriptome sequencing and analysis were conducted by OE Biotech Corporation Ltd. (Shanghai, China).

The libraries were sequenced on the Illumina HiSeq X Ten platform. Approximately 50 megabytes of raw reads for each sample were generated. Raw data were preprocessed using Trimmomatic. After removing the reads containing poly N and the low-quality reads, clean reads were obtained and mapped to the mouse genome (GRCm38.p6) using HISAT2. The fragments per kilobase of exon model per million mapped fragments (FPKM) value of each gene was calculated using Cufflinks, and the read counts of each gene were obtained by HTSeq-count. Differentially expressed genes (DEGs) were identified using the DESeq (2012) R package, followed by gene set enrichment analysis (GSEA). *P* value < 0.05 and fold change > 2 or fold change < 0.5 were set as the thresholds for significantly differential expression. To demonstrate the expression pattern of genes in different groups and samples, hierarchical cluster analysis of DEGs was performed. R software based on the hypergeometric distribution was employed for Gene Ontology (GO) enrichment and Kyoto Encyclopedia of Genes and Genomes (KEGG) pathway enrichment analysis of DEGs. Immune infiltration analysis was performed using Immune Cell Abundance Identifier (ImmuCellAI).

### 2.6 Statistical analysis

Data are presented as the mean ± standard error of the mean (SEM). The GraphPad Prism (version 8.0; GraphPad Software, San Diego, CA, USA) was utilized for statistical analyses. Normality of distribution was confirmed using the *Shapiro-Wilk test*, and differences among groups were assessed using the *Student’s t-test* or *two-way ANOVA* when appropriate, with the *Tukey’s post-hoc test*. The *log-rank test* was used to compare survival rates. *P* < 0.05 was considered statistically significant.

## 3 Results

### 3.1 Tacrolimus ameliorated colitis in a DSS mouse model

**Figure 1:**
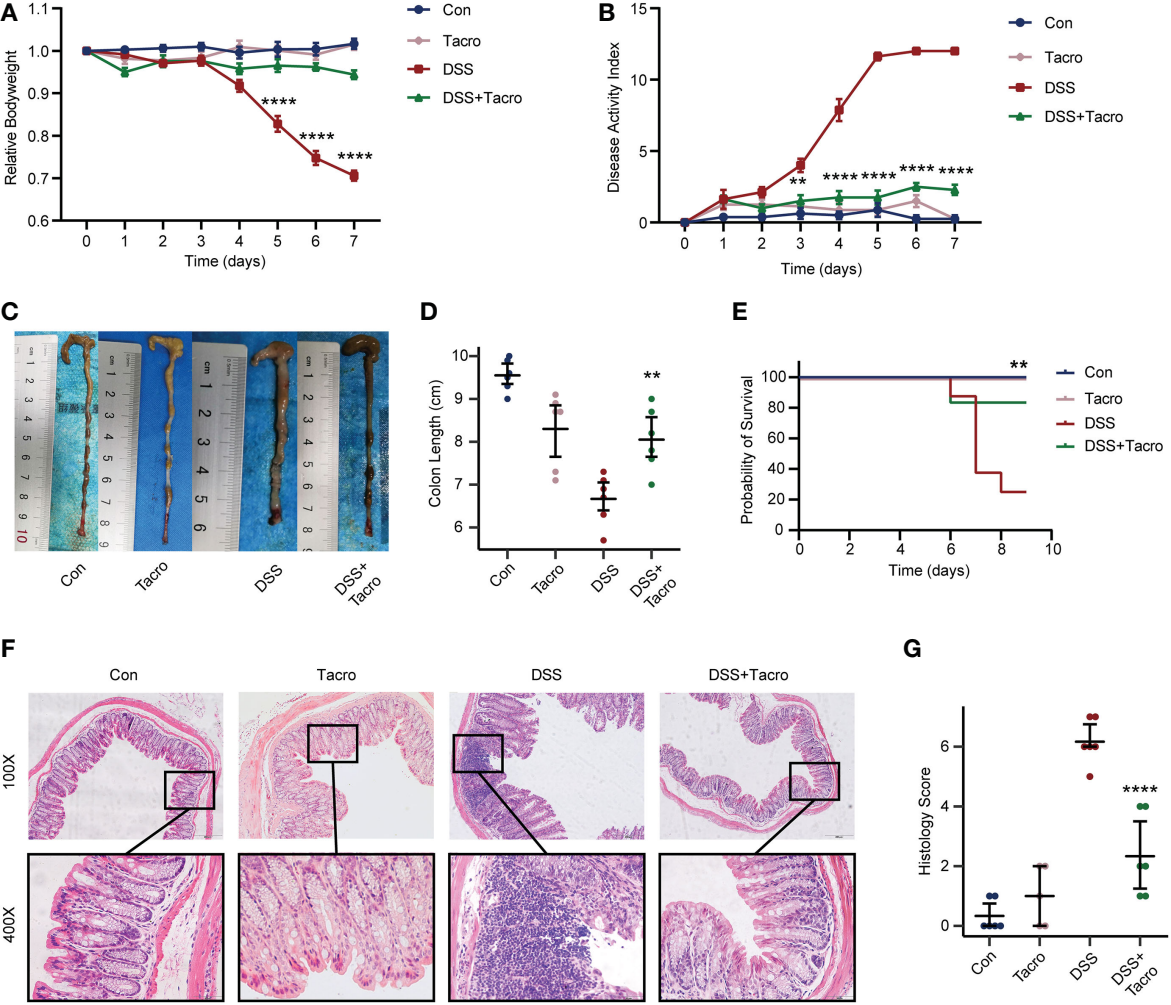
Tacrolimus alleviated DSS-induced colitis in mice. **(A, B)** Body weight changes and DAI scores of mice in different treatment groups. n=8 per group. **(C, D)** Gross view and length of colon. n=6 per group. **(E)** Survival rate of mice during the experiments. **(F)** Representative hematoxylin and eosin-stained images (100× and 400×) of colon sections. Scale bars, 200 μm (above) and 50 μm (below). **(G)** Histological scores of proximal and distal colons. n=6 per group. Data are presented as the mean ± SEM. **P < 0.01 and ****P < 0.0001. Significance reported for comparison of the DSS + Tacro and DSS groups. DAI, disease activity index; Con, control; DSS, dextran sulfate sodium; Tacro, 10 mg/kg tacrolimus.

Compared to the control mice, body weight loss, increased DAI scores and decreased survival rates were significantly observed in mice orally treated with 2.5% DSS for 7 days (**Figures 1A, B, E**). The dosage of tacrolimus (10 mg/kg) was determined by our previous study, which is the highest tolerated dose that most closely resembled the serum concentration in humans (Zhang et al., 2018). No significant difference in body weight, DAI scores, survival rate or colon length was observed in the tacrolimus and control groups, supporting the safety of tacrolimus at this dosage. Body weight, DAI scores and survival rates in the DSS colitis model were significantly (*P*<0.0001, *P*<0.0001, *P*<0.01, respectively) ameliorated by tacrolimus treatment (**Figures 1A, B, E**). Furthermore, colitis manifestations, including the shortened length, disruption of crypt-villus structure, goblet cell loss and immune cell infiltration, as determined by morphological changes and pathological scores were significantly (*P*<0.01) ameliorated in the DSS + Tacro group compared to the DSS group (**Figures 1C, D, F, G**). All these results indicated that tacrolimus ameliorated DSS-induced colitis in both clinical manifestation and pathological changes.

Lower dosages of tacrolimus (0.1 mg/kg and 1 mg/kg) were also used in the acute DSS colitis model in our study. Tacrolimus at these lower dosages attenuated the increased DAI scores and improved survival in colitis (**Supplementary Figures 1B, E**), whereas no significant differences in weight change and colon length were observed in the low-dose DSS + Tacro group compared to the DSS group (**Supplementary Figures 1A, C, D**). These data also supported the effectiveness of tacrolimus in the DSS mouse model. For the consistency and effectiveness of the study, tacrolimus was used at a dosage of 10 mg/kg in the following study.

### 3.2 Tacrolimus induced beneficial alterations of the gut microbiome in DSS-induced colitis, characterized by a remarkable expansion of the genus Lactobacillus

**Figure 2:**
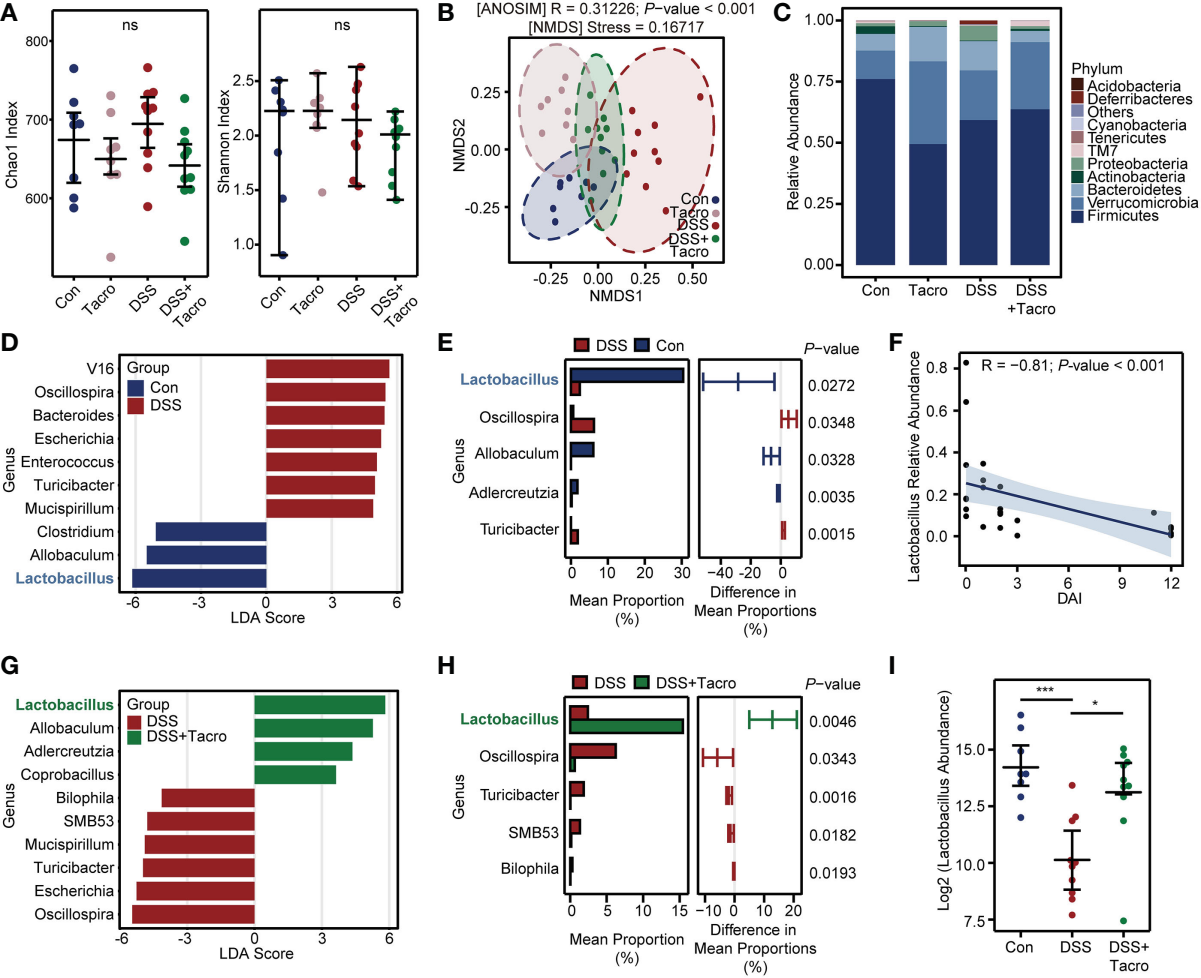
Tacrolimus triggered a significant increase in the genus *Lactobacillus* in colitis. **(A)** The microbial α-diversity in the cecal contents of mice. **(B)** β-diversity analysis based on the NMDS and ANOSIM methods. **(C)** Relative abundance of gut bacterial composition at the phylum level. **(D)** The LDA scores between the control and DSS-treated groups at the genus level. The threshold was set as 3. **(E)** The significantly different bacteria between the control and DSS-treated groups evaluated by STAMP. **(F)** Correlation analysis between *Lactobacillus* relative abundance and DAI score. A total of 28 samples from the Con, DSS and DSS + Tacro groups were included in this analysis. **(G)** The LDA scores of the DSS + Tacro group when compared with the DSS group at the genus level. **(H)** Significantly different genera between the DSS and DSS + Tacro groups evaluated by STAMP. **(I)** *Lactobacillus* abundance in log 2 scale among different treatment groups. Data are presented as the mean ± SEM. *P < 0.05, ***P < 0.001, ns, no significance; Con, control; DSS, dextran sulfate sodium; Tacro, 10 mg/kg tacrolimus; NMDS, nonmetric multidimensional scaling; LDA, linear discriminant analysis; STAMP, statistical analysis of metagenomic profile; DAI, disease activity index.

To investigate the effect of tacrolimus on the diversity and composition of the gut microbiome in DSS-treated mice, 16S rRNA amplicon sequencing of DNA extracted from the cecal contents of mice was performed. The α-diversity of gut microbiota was not significantly different among the Control, Tacro, DSS and DSS + Tacro groups, manifested as the Chao1 index or Shannon index in **Figure 2A**.

According to the β-diversity of the gut microbiota, NMDS analysis was used, and there was significant (*P*<0.001) separation of microbial communities among the control, DSS and DSS + Tacro groups. The DSS group was located distant from the control group, while the position of the DSS + Tacro group was closer to the control group than to the DSS group (**Figure 2B**). Consistent with a previous study (Shin et al., 2015), DSS administration resulted in an obvious increase in the relative abundance of pylum_*Proteobacteria* and *Deferribacteres* and a decrease in the *Firmicutes/Bacteroidetes* ratio. All these changes were reversed by tacrolimus treatment (**Figure 2C**). LEfSe is a commonly used method to identify potential biomarkers with significant differences among groups. In our study, the abundances of the genera_*Oscillospira, Bacteroides, Escherichia, Enterococcus*, *Turicibacter and Mucispirillum* were significantly enriched, whereas the abundances of the genera_*Lactobacillus, Allobaculum* and *Clostridium* were significantly decreased in the DSS group compared to the normal control group (**Figure 2D**). Among the bacteria that differed significantly between the control and DSS groups at the genus level, *Lactobacillus* occupied the largest mean proportion, approximately 30% (**Figure 2E**). Correlation analysis showed that the DAI scores of colitis were negatively correlated with the abundance of the genus_*Lactobacillus*, as shown in **Figure 2F** (R=-0.81; *P* < 0.001) and **Supplementary Figure 2A** (R=-0.76; *P* < 0.001).

In the DSS + Tacro group, LEfSe analysis found that the increased abundance of the genera_*Oscillospira, Escherichia, Turicibacter, Mucispirillum, SMB53, Bilophila* as well as the decreased abundance of the genera_*Lactobacillus* and *Allobaculum* in the colitis model were significantly reversed by tacrolimus treatment (**Figure 2G**). Tacrolimus remarkably (*P* < 0.05) restored the abundance of the genera_ *Lactobacillus, Oscillospira, Turicibacter*, etc. (**Figures 2H, I**). Among these significant changes in the microbiota, the abundance of the genus_*Lactobacillus* showed the most significant changes, with the highest proportion (approximately 15%) in the DSS + Tacro group (**Figure 2H**). To validate our findings, we performed the same research in another center and confirmed similar trends, both showing a decrease in the genus_*Lactobacillus* in the DSS group and a restoration of its abundance when treated with tacrolimus (**Supplementary Figures 2A, B**). This strengthened our confidence in the findings, although the composition of gut microbiota is not similar in two centers.

### 3.3 *Lactobacillus plantarum* 550 (*Lacto*) further ameliorated colitis combined with tacrolimus treatment

**Figure 3:**
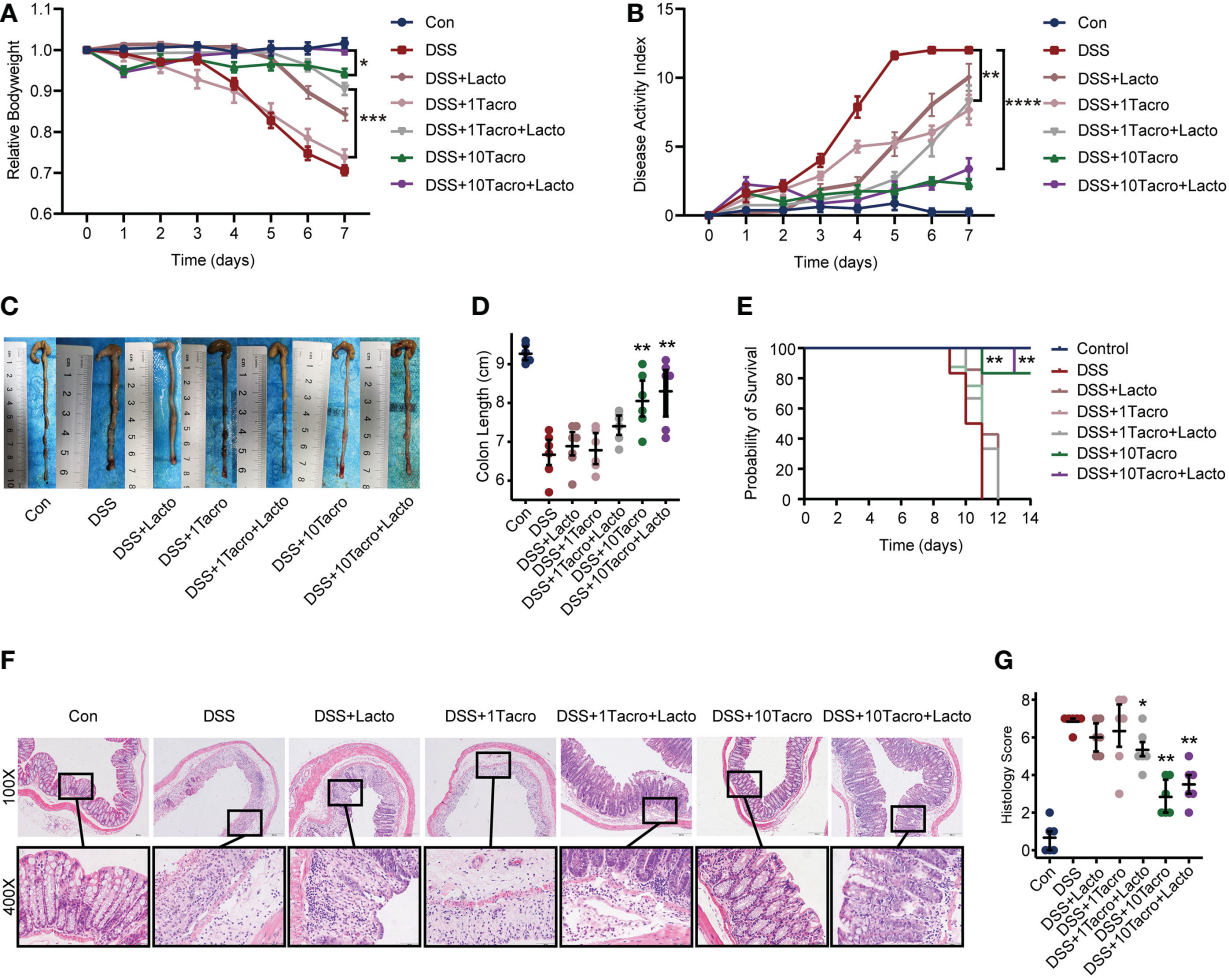
The combination of tacrolimus and *Lactobacillus plantarum* 550 achieved better remission in colitis. **(A, B)** Body weight changes and DAI scores of mice in different treatment groups. n=8 per group. **(C, D)** Gross view and length of colons. n=6 per group. **(E)** Survival rate of mice during the experiments. **(F)** Representative hematoxylin and eosin-stained images (100× and 400×) of colon sections. Scale bars, 200 μm (above) and 50 μm (below). **(G)** Histological scores of proximal and distal colons. n=6 per group. Data are presented as the mean ± SEM. *P < 0.05, **P < 0.01, ***P < 0.001 and ****P < 0.0001. The significance reported in **(B)**, **(D)**, **(E)**, and **(G)** is for comparison all to the DSS group. DAI, disease activity index; Con, control; DSS, dextran sulfate sodium; 1Tacro, 1 mg/kg tacrolimus; 10Tacro, 10 mg/kg tacrolimus; Lacto, *Lactobacillus plantarum* 550.

*Lacto*, a strain of the genus *Lactobacillus plantarum*, was recently isolated from pickle of Sichuan cuisine by our group and determined by 16S ribosomal DNA identification. Body weight changes in the DSS + 1Tacro (1 mg/kg) group and the DSS + *Lacto* group were not significantly different from those in the DSS group, whereas the combination of *Lacto* in the DSS + 1Tacro + Lacto group maintained the body weight at a higher level, even closer to that in the DSS + 10Tacro (10 mg/kg) group (**Figure 3A**). Moreover, *Lacto* further prevented body weight loss in DSS + 10Tacro mice (**Figure 3A**) compared to monotherapy with tacrolimus in the DSS model (*P* < 0.05). As determined by DAI, the combination of *Lacto* with either tacrolimus at doses of 1 mg/kg or 10 mg/kg significantly (*P* < 0.01 and *P* < 0.0001, respectively) decreased DAI scores compared to the DSS group (**Figure 3B**). Colon length, which is negatively related to the inflammation of colitis (Liu et al., 2017), was measured, and a significantly increased length was reported in the DSS + 10Tacro (*P* < 0.01) as well as the DSS + Tacro + Lacto groups (*P* < 0.05, *P* < 0.01, respectively), with the most increased length in the DSS + 10 Tacro + Lacto group (**Figures 3C, D**). Survival rates were significantly (*P* < 0.01) increased in the DSS + 10Tacro and DSS + 10Tacro + Lacto groups, with the latter group showing better survival (**Figure 3E**). Compared to the DSS group, cotreatment with tacrolimus and *Lacto* obviously (*P* < 0.05 and *P* < 0.01, respectively) relieved the inflammation of the colonic mucosa, as determined by the histological scores (**Figures 3F, G**). Overall, *Lacto*, combined with tacrolimus, further ameliorated colitis in a mouse model in clinical and histological manifestations.

### 3.4 The combination of tacrolimus and lacto significantly inhibited the proinflammatory signaling pathways

**Figure 4:**
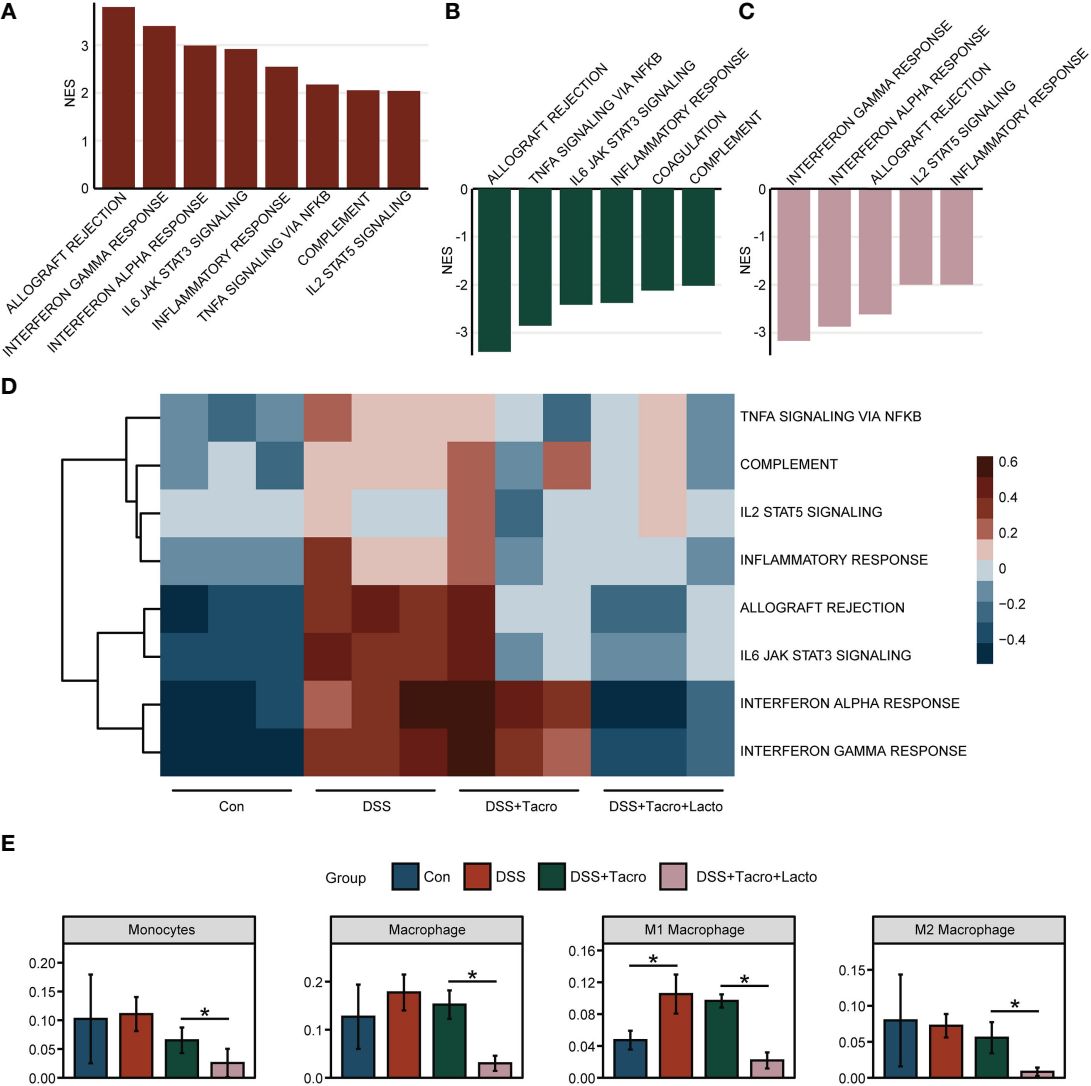
*Lactobacillus plantarum* 550 supplementation modulated intestinal mucosa inflammation at the transcriptional level. **(A–C)** Gene set enrichment analysis. NES values greater than 2 were demonstrated. The results represent pathways modulated in the **(A)** DSS group versus Con group, **(B)** DSS + Tacro group versus DSS group and **(C)** DSS + Tacro + Lacto group versus DSS + Tacro group. **(D)** Results of gene set variation analysis for selected biological processes in all groups. **(E)** The profile of infiltrating immune cells in the Con, DSS, DSS + Tacro and DSS + Tacro + Lacto groups. Data are presented as the mean ± SEM. *P < 0.05. NES, normalized enrichment score; Con, control; DSS, dextran sulfate sodium; Tacro, 10 mg/kg tacrolimus; Lacto, *Lactobacillus plantarum* 550. n=3 per group.

RNA sequencing data revealed that at the transcriptional level, the immune and inflammation-related signaling pathways, including the allograft rejection, IFN-γ response, IFN-α response, IL6 JAK STAT3 signaling, inflammatory response, TNFα signaling *via* NF-κB, complement and IL2 STAT5 signaling were significantly upregulated after DSS administration (**Figure 4A**). Most of these pathways, including the allograft rejection, TNFα signaling *via* NF-κB, IL6 JAK STAT3 signaling, inflammatory response and complement were significantly downregulated by tacrolimus (**Figure 4B**). When tacrolimus was combined with *Lacto*, the signaling pathways, including IFN-γ and IFN-α response, allograft rejection, IL2 STAT5 signaling and the inflammatory response, were further downregulated (**Figure 4C**), implying that the combined therapy of tacrolimus and *Lacto* further inhibited the inflammatory response in the colonic mucosa of colitis mice compared to the tacrolimus monotherapy group. The results of the gene set variation analysis are visualized in **Figure 4D**. The range of colors indicates the relative abundance of the signaling pathways mentioned above for each sample.

To demonstrate the profile of immune cells in the colonic mucosa, we analyzed the immune cell infiltration levels based on transcriptome sequencing data. As shown in **Figure 4E**, the proportion of monocytes and macrophages, especially the M1 subtypes, was elevated in the DSS group, while the M2 subtypes were slightly reduced with DSS intervention, in accordance with published reports overviewing the opposing roles of M1 and M2 macrophages in DSS-induced colitis (Lin et al., 2014). To a certain extent, tacrolimus suppressed the increase in the proportion of monocytes and macrophages, whereas coadministration of tacrolimus and *Lacto* significantly (*P* < 0.05) inhibited the expansion of these innate immune cells (**Figure 4E**) but not other immune cells, including dendritic cells (DCs), type 1 conventional dendritic cells (cDC1), type 2 conventional dendritic cells (cDC2), plasmacytoid dendritic cells (pDCs), B cells, memory B cells, B1 cells, follicular B cells, germinal center B cells, CD8^+^ cytotoxic T cells (Tc), naïve CD8^+^ T cells, Tγδ cells (Tgd), mast cells, M2 macrophages, NK cells, basophils, granulocytes and eosinophils (**Supplementary Figure 3**). Altogether, our results suggested that the downregulation of inflammatory signaling pathways may be one of the possible mechanisms accounting for the alleviation of colonic mucosal inflammation after *Lacto* supplementation.

### 3.5 Combination of tacrolimus and *Lacto* improved the diversity of the gut microbiome and changed the bile acids profiles in colitis

**Figure 5:**
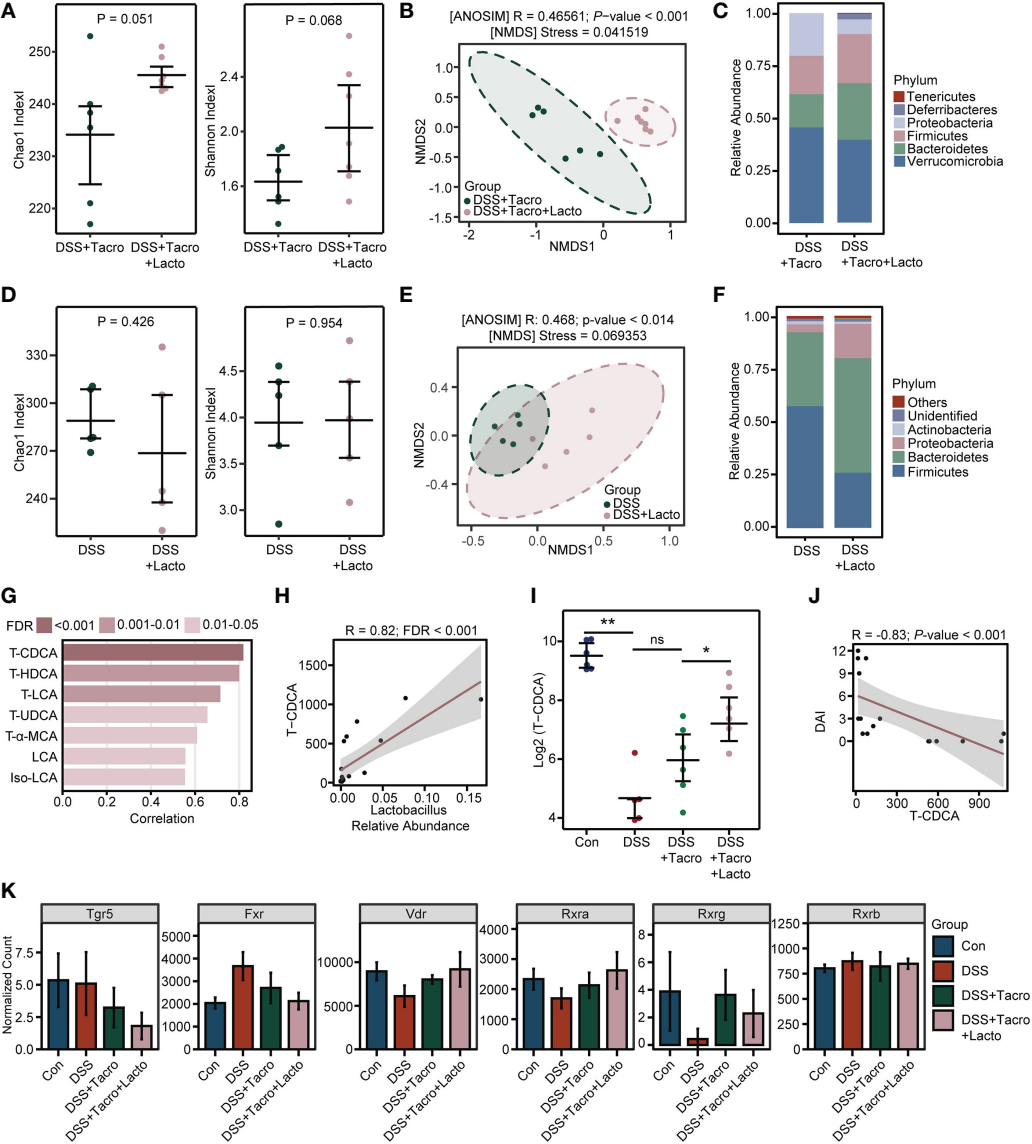
Tacrolimus coadministered with *Lactobacillus plantarum* 550 changed the diversity of the gut microbiome and bile acid metabolism in colitis. **(A, D)** Gut microbial α-diversity determined by Chao1 and Shannon indexes. **(B, E)** β-diversity analysis based on the NMDS and ANOSIM methods. **(C, F)** Relative abundance of gut bacterial composition at the phylum level. **(G, H)** Correlation analysis between bile acid concentrations and *Lactobacillus* relative abundance. **(I)** Relative concentration of TCDCA on a log 2 scale. **(J)** Correlation analysis between DAI score and TCDCA concentration. **(K)** The normalized expression levels of bile acid receptors among all groups. Data are presented as the mean ± SEM. *P < 0.05, **P < 0.01, ns, no significance. NMDS, nonmetric multidimensional scaling; FDR, false discovery rate; DAI, disease activity index; Con, control; DSS, dextran sulfate sodium; Tacro, 10 mg/kg tacrolimus; Lacto, *Lactobacillus plantarum* 550; TCDCA, taurochenodeoxycholic acid; Tgr5, Takeda G protein-coupled receptor 5; Fxr, farnesoid X receptor; Vdr, vitamin D (1,25- dihydroxyvitamin D3) receptor; Rxra, retinoid X receptor alpha; Rxrg, retinoid X receptor gamma; Rxrb, retinoid X receptor beta.

Next, to explore whether the effect of *Lacto* supplementation on colitis remission had some relationship with alterations in the gut microbiome, similar to that previously observed in the tacrolimus-treated group (Zhang et al., 2018; Jiao et al., 2020), we compared the results of 16S rRNA sequencing in the presence and absence of *Lacto*. Increased α-diversity of the gut microbiome was observed in the combination treatment group compared to the tacrolimus-treated group, as reflected by the Chao1 and Shannon indexes (**Figure 5A**). The results from NMDS and ANOSIM analysis clearly indicated that the bacterial communities of these two groups differed from each other (**Figure 5B**). In contrast with tacrolimus treatment, the relative abundance of the pathogenic bacteria *Proteobacteria* was further reduced, while the abundance of *Firmicutes* and *Bacteroidetes*, the two major phyla composing the gut microbiota community in healthy humans, was better maintained by *Lacto* supplementation (**Figure 5C**). However, *Lacto* alone did not significantly affect the bacterial diversity or cause beneficial changes in the composition of gut microbiota in colitis (**Figures 5D–F**).

Bile acid metabolism is a biological process in which specific intestinal floras are actively involved (Wahlström et al., 2016). Recently, gut microbiome-derived bile acids have been reported to play a critical role in IBD patients and experimental colitis(Lavelle and Sokol, 2020; Sinha et al., 2020; Thomas et al., 2022). Considering the significant changes in diversity and composition after the combination of tacrolimus and *Lacto*, bile acids were also detected in our study through high-throughput sequencing. Among all the bile acids analyzed in the Con, DSS and DSS + Tacro groups, the cecal taurochenodeoxycholic acid (TCDCA) level was most strongly correlated with the relative abundance of the genus_*Lactobacillus* (**Figure 5G**). Furthermore, a significant positive correlation (R=0.82; FDR<0.001) between the TCDCA concentration and the abundance of genus_*Lactobacillus* was observed in our experiments (**Figure 5H**).

The TCDCA levels that remarkably (*P* < 0.01) declined after DSS intervention were not restored by tacrolimus treatment. However, the combined treatment of tacrolimus plus *Lacto* drastically (*P* < 0.05) increased the TCDCA concentrations in comparison with tacrolimus treatment (**Figure 5I**). In addition, the cecal TCDCA concentrations of mice were negatively (R=-0.83; *P*<0.001) correlated with the DAI score (**Figure 5J**), suggesting an association between this bile acid and colitis severity.

Bile acids can function as signaling molecules to activate their receptors, thereby affecting intestinal inflammation (Perino et al., 2021; Yang et al., 2021). As presented by our transcriptome analysis, DSS administration perturbed the expression of the bile acid-sensing receptors, including G-protein-coupled bile acid receptor 1 (Gpbar1, TGR5), farnesoid X receptor (FXR), vitamin D (1,25-dihydroxyvitamin D3) receptor (VDR), and retinoid X receptor (RXR), to various degrees, all of which were mitigated somewhat by tacrolimus treatment except TGR5, and this effect was amplified further by coadministration of *Lacto* (**Figure 5K**). Collectively, our findings demonstrated that the combination of tacrolimus and *Lacto* improved the diversity and composition of the gut microbiome as well as bile acid metabolism in colitis mice, which possibly contributed to the alleviation of intestinal inflammation. The detailed mechanisms remain to be further explored.

## 4 Discussion

In this study, we confirmed that tacrolimus ameliorated DSS-induced colitis in a mouse model. Beneficial alterations of the gut microbiome characterized by a remarkable expansion of the genus *Lactobacillus* were observed after tacrolimus treatment. Oral supplementation with one of the species in this genus (*Lactobacillus plantarum* 550, *Lacto*) further ameliorated colitis. Compared to the monotherapy of tacrolimus, the combination of *Lacto and* tacrolimus further downregulated the proinflammatory signaling pathways while significantly inhibiting the proportion of monocytes and macrophages, especially the M1 subtypes in the colon. During this process, the diversity of the gut microbiome as well as bile acid metabolism was significantly changed, which may contribute to the remission of colitis.

Recently, tacrolimus has been recommended for the treatment of refractory IBD, showing efficacy in both human and animal colitis (Baumgart et al., 2006; Yoshino et al., 2010). In addition to the traditional calcineurin-dependent inhibitory effect on T cells, several studies have reported that the mechanism of tacrolimus-ameliorated colitis might be related to the suppression of activated macrophages (Yoshino et al., 2010; Cai et al., 2021), the restriction of dendritic cell migration and the subsequent differentiation of CD4+ T cells to Th1 and Th17 cells (Regmi et al., 2019), as well as protection against apoptosis-mediated intestinal epithelial injury (Satake et al., 2022). In accordance with previous research, we found a significant alleviation of DSS-induced colitis by tacrolimus treatment. Signaling pathways, including the IFN-γ response, TNFα signaling *via* NF-κB, IL6 JAK STAT3 signaling and inflammatory response pathways upregulated after DSS administration were significantly inhibited by tacrolimus.

The gut microbiome plays an important role in the regulation of the immune response (Shi et al., 2017). Except for the immunosuppressive effect of tacrolimus itself, previous studies by our group and other groups have reported that tacrolimus changes the diversity and composition of the gut microbiome, resulting in the regulation of graft rejection (Jiang et al., 2018; Zhang et al., 2018), glucose metabolism (Bhat et al., 2017; Jiao et al., 2020), infections (Tourret et al., 2017) and endothelial function (Toral et al., 2018). In this study, the changes in the tacrolimus-induced microbiome in a colitis model were first described, which was characterized by a significant increase in the abundance of *Lactobacillus* followed by *Allobaculum* at the genus level. This is consistent with our prior studies showing the abundant genera *Lactobacillus* and *Allobaculum* after tacrolimus treatment in normal mice (Zhang et al., 2018; Jiao et al., 2020). *Allobaculum* was favored as an indicator of defective immune responses (Dimitriu et al., 2013).

The genus *Lactobacillus* was commonly reported to be decreased in IBD and experimental colitis (Sartor, 2004; Wang et al., 2020), which was also confirmed in our present study. And restoration or supplementation of this probiotic was associated with the alleviation of intestinal inflammation (Oliva et al., 2012; Wang et al., 2020). Based on our previous research proposing that tacrolimus plus gut microbiota achieved an increased allograft survival rate (Zhang et al., 2018), this study confirmed that the combination of tacrolimus and *Lactobacillus* strains played a positive role in colitis. Since we observed that the colonic contents were inadequate for the detection of two-omics, especially in the condition of colitis, the cecal content was used following the protocol as previous studies (Wong et al., 2022). There are certain limitations to do this since the most representative alterations in the gut microbiome are in the area where inflammation occurs. However, the efficacy of combination therapy confirmed the decreased abundance of the genus_*Lactobacillus* in DSS-induced colitis model.

Efficacy of tacrolimus combined with *Lactobacillus* spp. has been demonstrated in lupus-prone mice (Kim et al., 2021), graft-versus-host disease (Beak et al., 2022), and adult-type atopic dermatitis (Moroi et al., 2011), which acted by modulating the balance of Treg cells and Th17 cells. Here, we chose *Lacto*, one strain recently isolated from pickle by our group in Sichuan cuisine and proven to generate short-chain fatty acids (butyric acid, acetic acid, propionic acid, etc.) in preliminary experiments as a supplement for tacrolimus treatment. In recent years, some strains from *Lactobacillus plantarum* were reported to attenuate colitis in mice by restoring the disturbed gut microbiota, affecting intestinal barrier functions and immunity-related gene expression(Sun et al., 2020; Wu et al., 2022). Our results showed better remission of colitis in the combination treatment group than in the tacrolimus monotherapy group, accompanied by further downregulation of inflammatory signaling pathways. As we showed in **Figure 3A**, the combination of tacrolimus and *Lacto*, at both lower dosage (1 mg/kg) and higher dosage (10 mg/kg) promoted the therapeutic effect of colitis in mice, compared to the monotherapy of tacrolimus. Especially, the efficacy of tacrolimus (1mg/kg) combined with *Lacto* was close to the tacrolimus (10mg/kg) monotherapy. All of these results have shown that the combination therapy may reduce the dosage of tacrolimus for the treatment of colitis, thereby reducing dose-related side effects. Furthermore, by analyzing transcriptome sequencing data, we first reported that tacrolimus combined with *Lacto* treatment decreased the proportion of both colonic monocytes and macrophages in colitis but not dendritic cells (DC), type 1 conventional dendritic cells (cDC1), type 2 conventional dendritic cells (cDC2), plasmacytoid dendritic cells (pDC), B cells, memory B cells, B1 cells, follicular B cells, germinal center B cells, CD8^+^ cytotoxic T cells (Tc), naïve CD8^+^ T cells, Tγδ cells (Tgd), mast cells, M2 macrophages, NK cells, basophils, granulocytes and eosinophils. Our data demonstrated that macrophages and the proinflammatory signaling pathway play a vital role in the amelioration of colitis induced by tacrolimus combined with *Lacto*.

Theoretically, an improved composition of the gut microbiome and bile acid metabolism might help explain the mechanism of amelioration after combination therapy. A recent study has shown that one strain from *Lactobacillus casei* ameliorated DSS-induced colitis in mice by increasing taurine-conjugated bile acids, and the activation of FXR by the increased TCDCA might exhibit anti-inflammatory effects (Wong et al., 2022). This possibly resulted from the activation of FXR shifting the polarization of macrophages toward the anti-inflammatory M2 phenotype, which promoted IL-10 secretion and inhibited IFN-γ production (Fiorucci et al., 2018). Published evidence also supports that colonic VDR signaling upregulated by 1,25−dihydroxyvitamin D (1,25(OH)_2_D_3_) or microbial metabolites (bile acids, butyrate, etc.) contributed to the restoration of macrophage subtype balance, thus ameliorating colitis (Zhu et al., 2019; Battistini et al., 2020). Moreover, the nuclear receptor RXR can form heterodimers with VDR or the peroxisome proliferator–activated receptor γ (PPAR γ) (Kiss et al., 2013), which is highly expressed in the colon and can be activated by butyrate (Desreumaux et al., 2001; Litvak et al., 2018). Either VDR/RXR or RXR/PPAR γ heterodimer activation was proved to protect against colonic inflammation (Desreumaux et al., 2001; Zhu et al., 2019). In our study, the observed increase in TCDCA concentration after combination treatment might not be attributed to a direct metabolic effect of the genus *Lactobacillus* on TCDCA, since this probiotic is mainly involved in the deconjugation, 7α−dehydroxylation and esterification of bile acids(Jia et al., 2018). Instead, this might result from complex factors such as regulation of enterohepatic circulation or bacterial interactions that deserve further research. Based on our results and the studies mentioned above, we speculated that the combination treatment of tacrolimus and *Lacto* induced beneficial alterations in the gut microbiome as well as the derived bile acid profile, which led to an upregulated expression of bile acid receptors (FXR, VDR, RXR) in colonic macrophages, thereby restoring the balance of the macrophage M1/M2 subtype and inhibiting the release of proinflammatory cytokine. As a result, the inflammatory signaling pathways were downregulated, and colonic inflammation was relieved. Additional experiments *in vitro* that correlating TCDCA, intestinal inflammation and *Lactobacillus plantarum* should be conducted to explore the mechanism underlying the efficacy of the combination therapy in colitis in the future.

## 5 Conclusion

The narrow therapeutic window of tacrolimus requires a promising treatment strategy for IBD that reduces the side effects while enhancing its efficacy. In this study, we confirmed the efficacy of tacrolimus treatment and innovatively demonstrated alterations in the tacrolimus-induced gut microbiome in a colitis model. *Lactobacillus plantarum* promotes the therapeutic effect of tacrolimus in colitis, possibly resulting from alterations in the gut microbiome and bile acid profile, which lead to the maintenance of macrophage M1/M2 subtype balance. Our findings offer a prospective strategy to combine tacrolimus and *Lactobacillus* for the treatment of colitis patients.

## Data availability statement

The raw data of the 16S rRNA sequencing in this study is publicly available in the SRA database, accession number PRJNA940074.

## Ethics statement

The animal study was reviewed and approved by Animal Ethics Committees of Beijing Chao-Yang Hospital and the Animal Ethics Committee of West China Hospital of Sichuan University (Ref. No. 20211277A).

## Author contributions

WL and DZ contributed equally to this paper. WL performed the experiments and drafted the manuscript. DZ performed part of the experiment, analyzed the data, drew the figures and helped to revise the manuscript. TH, YL and LS provided ideas for writing and helped with the experiments and revision of the manuscript. ZL and XP isolated the Lactobacillus plantarum 550 and completed pre-experiments for strain performance testing. LL and YW designed and supervised this study. All authors contributed to the article and agree to be accountable for the content of the work.
