# Supplementary material for: Combination of Lactobacillus plantarum improves the effects of tacrolimus on colitis in a mouse model
Source: Front Cell Infect Microbiol. 2023 Mar 13;13:1130820. doi: 10.3389/fcimb.2023.1130820 (PMC10040537; doi:10.3389/fcimb.2023.1130820)
Supplement: Supplementary file 1 [file DataSheet_1.docx]

**Supplementary Table 1. The disease activity index (DAI) scores of colitis.**

| Score | Weight loss (%) | Stool consistency | Blood in feces |
| --- | --- | --- | --- |
| 0 | None | Firm or soft, dry stools | - or + |
| 1 | ≤5 | —— | —— |
| 2 | >5 and ≤10 | Loose, moist stools | ++ or +++ |
| 3 | >10 and ≤15 | —— | —— |
| 4 | >15 | Watery diarrhea | ++++ or gloss bleeding |

**Supplementary Table 2.** **Bile acids identified in this study.**

| Targeted bile acids | | | | Abbreviations | Targeted bile acids | Abbreviations | |
| --- | --- | --- | --- | --- | --- | --- | --- |
| Dehydrolithocholic acid | | | | DHLCA | Allocholic acid | alloCA |  |
| Isoallolithocholic acid | | | | isoalloLCA | Cholic acid | CA |  |
| Isolithocholic acid | | | | isoLCA | Glycolithocholic acid | GLCA |  |
| Lithocholic acid | | | | LCA | Glycoursodeoxycholic  acid | GUDCA |  |
| 23-Nordeoxycholic acid | | | 23-NDCA | | Glycohyodeoxycholic acid | GHDCA |  |
| 7-Ketolithocholic acid | | 7-KLCA | | | Glycodeoxycholic acid | GDCA |  |
| 12-Ketolithocholic acid | | | 12-KLCA | | Glycohyocholic acid | GHCA |  |
| Apocholic acid | | | | apoCA | Glycocholic acid | GCA |  |
| Ursodeoxycholic acid | | | | UDCA | Taurolithocholic acid | TLCA |  |
| Hyodeoxycholic acid | | | | HDCA | Tauroursodeoxycholic acid | TUDCA |  |
| Chenodeoxycholic acid | | | | CDCA | Taurohyodeoxycholic acid | THDCA |  |
| Deoxycholic acid | | | | DCA | Taurochenodeoxycholic acid | TCDCA |  |
| Isodeoxycholic acid | | | | isoDCA | Taurodeoxycholic acid | TDCA |  |
| Dehydrocholic acid | | | | DHCA | Tauro α-Muricholic acid | T-α-MCA |  |
| 7,12-Diketolithocholic acid | | | | 7,12-DKLCA | Tauro β-Muricholic acid | T-β-MCA |  |
| 6,7-Diketolithocholic acid | | | 6,7-DKLCA | | Taurocholic acid | TCA |  |
| 7-Ketodeoxycholic acid | 7-KDCA | | | | Hyocholic acid | HCA |  |
| 12-Dehydrocholic acid | | | 12-DHCA | |  |  |  |
| 3-Dehydrocholic acid | 3-DHCA | | | |  |  |  |
| Ursocholic acid | | | | UCA |  |  |  |
| α-Muricholic acid | | | | α-MCA |  |  |  |
| β-Muricholic acid | | | | β-MCA |  |  |  |


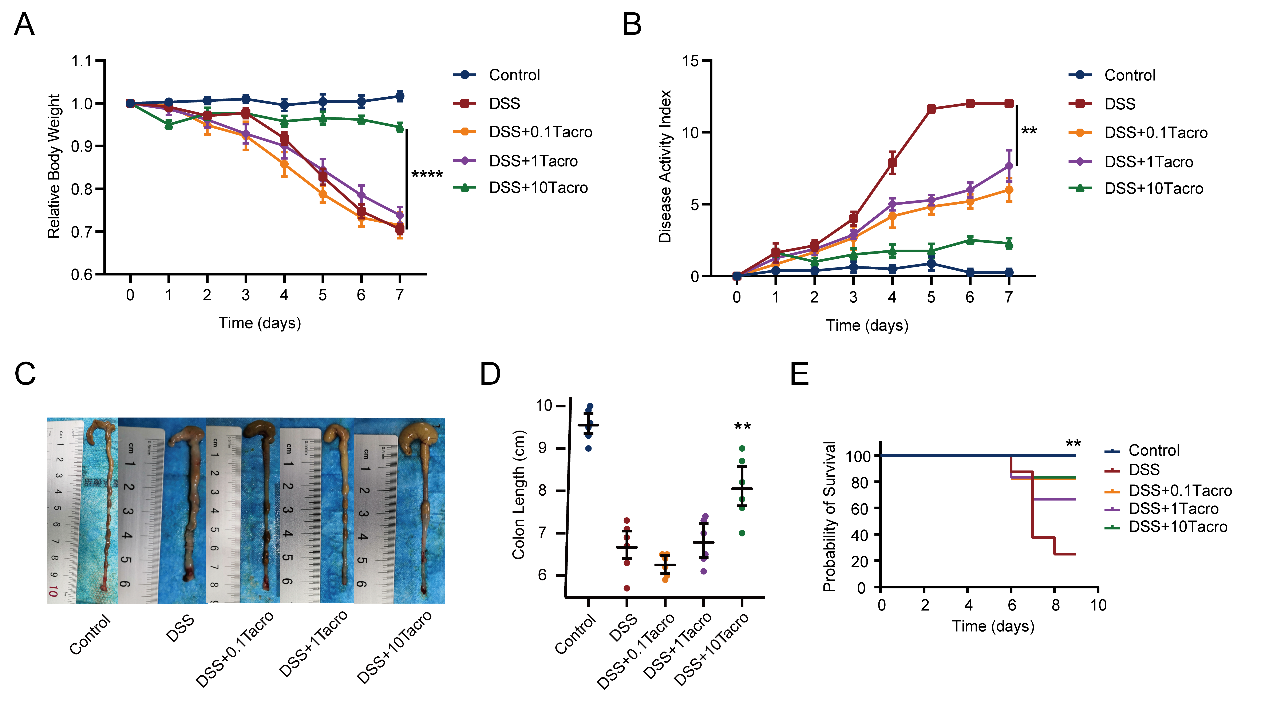


**Supplementary Figure 1. Tacrolimus treatment at different dosages in DSS-induced colitis.** (A&B) Body weight changes and DAI scores of mice in different treatment groups. n=6~8 per group. (C&D) Gross view and length of colon. n=6 per group. (E) Survival rate of mice during the experiments. Data are presented as the mean ± SEM. ***P* < 0.01 and **** *P* < 0.0001. Significance reported in (D) and (E) is for comparison of the DSS+10Tacro and DSS groups. Con: control; DSS: dextran sulfate sodium; 0.1Tacro: 0.1 mg/kg tacrolimus; 1Tacro: 1 mg/kg tacrolimus; 10Tacro: 10 mg/kg tacrolimus.


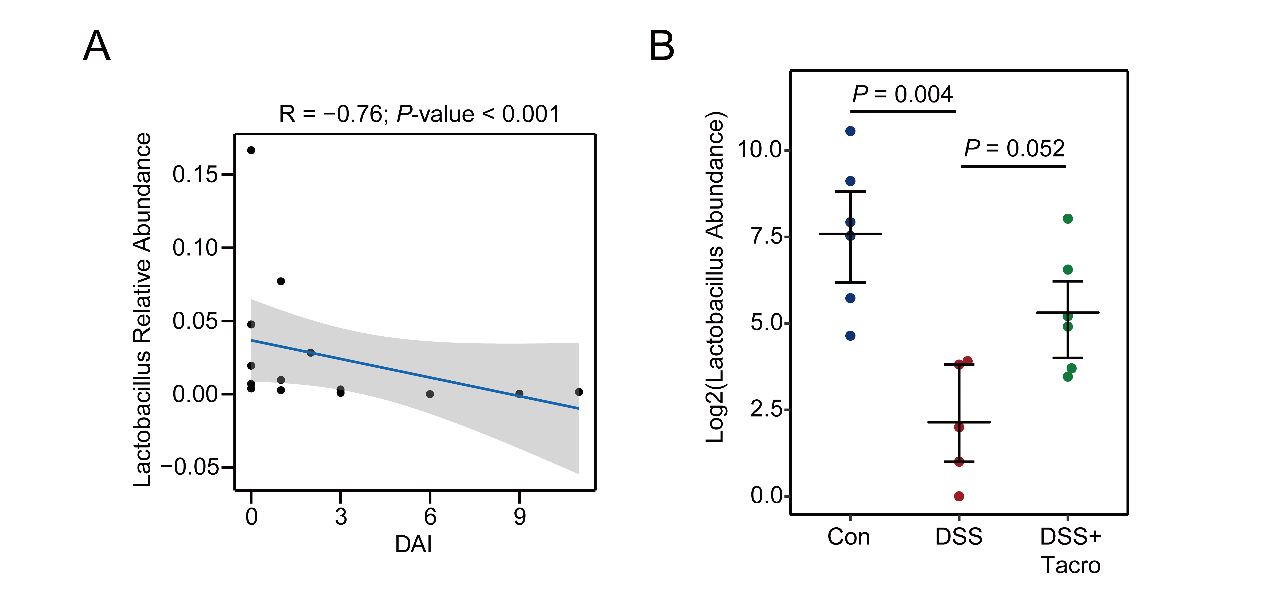


**Supplementary Figure 2. *Lactobacillus* levels in samples from West China Hospital.** (A) Correlation analysis between *Lactobacillus* relative abundance and DAI score. A total of 17 samples from the Con, DSS and DSS+Tacro groups were included in this analysis. (B) *Lactobacillus* abundance in log 2 scale among different treatment groups. Data are presented as the mean ± SEM. DAI: disease activity index. Con: control; DSS: dextran sulfate sodium; Tacro: 10 mg/kg tacrolimus.


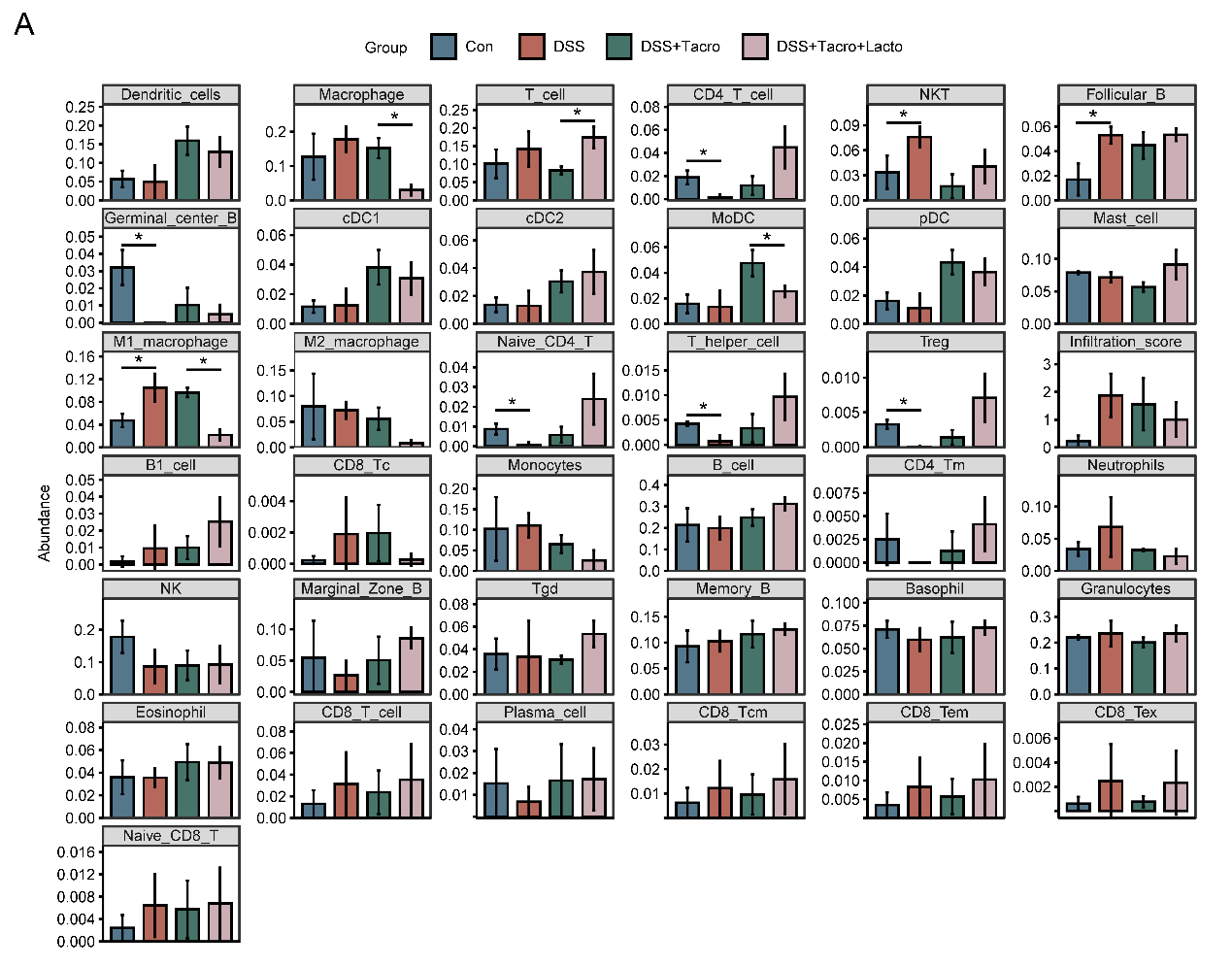


**Supplementary Figure 3. The profile of infiltrating immune cells among the Con, DSS, DSS + Tacro and DSS + Tacro + Lacto groups.** Data are presented as the mean ± SEM. * *P* < 0.05. Con: control; DSS: dextran sulfate sodium; Tacro: 10 mg/kg tacrolimus; Lacto: *Lactobacillus plantarum* 550. n=3 per group.
